# 4125 Plan for a Retrospective Evaluation of a Multi-Modal Weight-centric Prediabetes Intervention.

**DOI:** 10.1017/cts.2020.136

**Published:** 2020-07-29

**Authors:** Raoul J Manalac, Tiffany Stewart, Donna Ryan

**Affiliations:** 1 Pennington Biomedical Research Center

## Abstract

OBJECTIVES/GOALS: To determine if a multi-modal, interdisciplinary intervention delivered to a group of prediabetic patients will result in reduced rates of diabetes progression. This project is a retrospective evaluation that will exam the feasibility and possibly efficacy of this intervention. METHODS/STUDY POPULATION: We will evaluate outcomes of 50 participants for the clinic, aged 21-60 inclusive. Patients will have a Body Mass Index >25kg/m^2^ with a diagnosis of prediabetes. Patients must be non-pregnant, using approved contraception, and agree to not become pregnant for 1 year after enrollment. After enrollment, the initial treatment period is for 1 year and includes a 12 week low calorie diet plan, a 6-month intensive behavioral and lifestyle modification plan followed by a 6 month behavior reinforcement extension. Weight management medications may be used if appropriate for the patient from a clinical perspective during the 6-month intensive behavioral/lifestyle modification. RESULTS/ANTICIPATED RESULTS: It is anticipated that there will be decreased weight with a mean weight loss goal of approximately >10%. Furthermore, it is expect that there will be improvement of other markers of metabolic disease. These include improvement of lipid values (LDL-C, HDL-C, Triglycerides, Total Cholesterol) as well as blood pressure with expected blood pressures of below 130/80 in greater than 50% of participants. Finally, It is expected that 50% or greater participants will have improvement of glycemic control. It is anticipated that greater than 50% of participants will have improvement of glycemic control and achieve normoglycemia. These values will be determined based upon fasting glucose or A1c. DISCUSSION/SIGNIFICANCE OF IMPACT: The significance of this intervention is enormous. By demonstrating feasibility in this trial, we can work toward both assessing efficacy and possibly dissemination of this model program. If these interventions provide durable changes at scale, this could help slow the epidemic of obesity and obesity related comorbid conditions.

